# *trans*-Dichloridobis(4-methoxy­aniline-κ*N*)palladium(II)

**DOI:** 10.1107/S1600536809018509

**Published:** 2009-05-23

**Authors:** Volodimir Bon, Svitlana Orysyk, Vasyl Pekhnyo

**Affiliations:** a Institute of General and Inorganic Chemistry, NAS Ukraine, Kyiv, prosp. Palladina 32/34, 03680 Ukraine

## Abstract

In the title compound, [PdCl_2_(C_7_H_9_NO)_2_], the Pd atom is situated on a crystallographic centre of inversion. The coordination environment of the Pd atom shows a slightly distorted square-planar geometry. The crystal structure exhibits weak inter­molecular Pd⋯Cl inter­actions, with Pd⋯Cl distances of 3.6912 (6) Å. A chain-like arrangement of mol­ecules realized by inter­molecular N—H⋯Cl hydrogen bonds is observed along [010].

## Related literature

For catalytic activity of Pd complex compounds, see: Ojwach *et al.* (2007 ▶). For anti­tumoral properties of Pd compounds, see: Casas *et al.* (2008 ▶). For related structures, see: Bon *et al.* (2009 ▶); Pan *et al.* (2006 ▶).
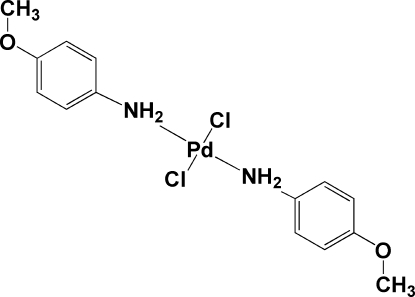


## Experimental

### 

#### Crystal data

[PdCl_2_(C_7_H_9_NO)_2_]
*M* *_r_* = 423.60
Monoclinic, 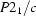

*a* = 4.7333 (1) Å
*b* = 6.0071 (1) Å
*c* = 27.6918 (5) Å
β = 94.806 (1)°
*V* = 784.60 (3) Å^3^
*Z* = 2
Mo *K*α radiation
μ = 1.53 mm^−1^
*T* = 173 K
0.25 × 0.08 × 0.04 mm

#### Data collection

Bruker APEXII CCD diffractometer
Absorption correction: numerical (*SADABS*; Bruker, 2005 ▶) *T*_min_ = 0.701, *T*_max_ = 0.941
4837 measured reflections
1577 independent reflections
1326 reflections with *I* > 2σ(*I*)
*R*_int_ = 0.024

#### Refinement

*R*[*F* ^2^ > 2σ(*F* ^2^)] = 0.023
*wR*(*F* ^2^) = 0.049
*S* = 1.04
1577 reflections
106 parameters
H atoms treated by a mixture of independent and constrained refinement
Δρ_max_ = 0.31 e Å^−3^
Δρ_min_ = −0.39 e Å^−3^

### 

Data collection: *APEX2* (Bruker, 2005 ▶); cell refinement: *SAINT* (Bruker, 2005 ▶); data reduction: *SAINT*; program(s) used to solve structure: *SHELXS97* (Sheldrick, 2008 ▶); program(s) used to refine structure: *SHELXL97* (Sheldrick, 2008 ▶); molecular graphics: *SHELXTL* (Sheldrick, 2008 ▶); software used to prepare material for publication: *publCIF* (Westrip, 2009 ▶).

## Supplementary Material

Crystal structure: contains datablocks I, global. DOI: 10.1107/S1600536809018509/im2115sup1.cif

Structure factors: contains datablocks I. DOI: 10.1107/S1600536809018509/im2115Isup2.hkl

Additional supplementary materials:  crystallographic information; 3D view; checkCIF report

## Figures and Tables

**Table 1:** Hydrogen-bond geometry (Å, °)

*D*—H⋯*A*	*D*—H	H⋯*A*	*D*⋯*A*	*D*—H⋯*A*
N1—H1*A*⋯Cl1^i^	0.85 (3)	2.53 (3)	3.353 (2)	162 (2)

Symmetry code: (i) 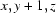.

**Fig. 1.**
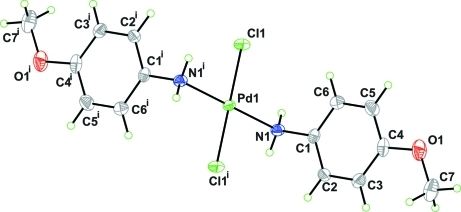
The title compound showing 50% probability displacement ellipsoids for the non-hydrogen atoms [Symmetry code: (i) -x, -y, -z].

**Fig. 2.**
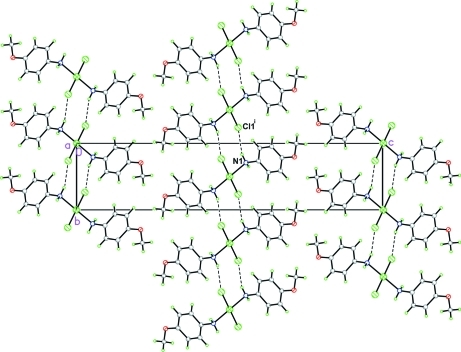
Crystal packing of title compound, projection down the a axis. Dashed lines indicate hydrogen bonds [Symmetry code: (i) x, y + 1, z].

## supplementary crystallographic information

### Comment

Coordination compounds of Pd with N-containing organic ligands attract considerable interest due to their antitumoral and catalytic activity (Casas *et al.*, 2008; Ojwach *et al.*, 2007). Similar structures with respect to the title compound differing in terms of the position of substituents at the aromatic ring were published earlier (Pan *et al.*, 2006; Bon *et al.*, 2009). The asymmetric unit of the title compound contains one-half of the molecule because Pd occupies a special position on the crystallographic centre of inversion. Pd shows a slightly distorted square-planar geometry of the coordination environment containing two chlorine atoms and two amino groups in *trans* position (Fig. 1). The crystal structure shows weak intermolecular Pd···Cl interactions with Pd—Cl distances of 3.6912 (6) Å. A chain-like arrangement of molecules realized by weak N—H···Cl hydrogen bonds is observed along *010* direction (Fig. 2; Table 1).

### Experimental

The yellow plate shaped crystals of the title compound were grown by slow evaporation of 10 ml of an ethanolic solution containing a mixture of 0.01 *M* H_2_[PdCl_4_] and 4-methoxyaniline in a 1:2 molar ratio 1:2.

### Refinement

H atoms bonded to N were located in a difference map and refined freely. Other H atoms were positioned geometrically and refined using a riding model with C—H = 0.98 Å for CH_3_ [*U*_iso_(H) = 1.5Ueq(C)] and C—H = 0.95 Å for CH [*U*_iso_(H) = 1.2Ueq(C)]

### Crystal data

**Table:**

[PdCl_2_(C_7_H_9_NO)_2_]	*F*(000) = 424
*M**_r_* = 423.60	*D*_x_ = 1.793 Mg m^−^^3^
Monoclinic, *P*2_1_/*c*	Mo *K*α radiation, λ = 0.71073 Å
Hall symbol: -P 2ybc	Cell parameters from 2257 reflections
*a* = 4.7333 (1) Å	θ = 3.0–26.4°
*b* = 6.0071 (1) Å	µ = 1.53 mm^−^^1^
*c* = 27.6918 (5) Å	*T* = 173 K
β = 94.806 (1)°	Plate, yellow
*V* = 784.60 (3) Å^3^	0.25 × 0.08 × 0.04 mm
*Z* = 2	

### Data collection

**Table:**

Bruker APEXII CCD diffractometer	1577 independent reflections
Radiation source: fine-focus sealed tube	1326 reflections with *I* > 2σ(*I*)
graphite	*R*_int_ = 0.024
Detector resolution: 8.26 pixels mm^-1^	θ_max_ = 26.4°, θ_min_ = 1.5°
φ and ω scans	*h* = −2→5
Absorption correction: numerical (*SADABS*; Bruker, 2005)	*k* = −7→7
*T*_min_ = 0.701, *T*_max_ = 0.941	*l* = −34→34
4837 measured reflections	

### Refinement

**Table:**

Refinement on *F*^2^	Primary atom site location: structure-invariant direct methods
Least-squares matrix: full	Secondary atom site location: difference Fourier map
*R*[*F*^2^ > 2σ(*F*^2^)] = 0.023	Hydrogen site location: inferred from neighbouring sites
*wR*(*F*^2^) = 0.049	H atoms treated by a mixture of independent and constrained refinement
*S* = 1.04	*w* = 1/[σ^2^(*F*_o_^2^) + (0.0205*P*)^2^ + 0.3835*P*] where *P* = (*F*_o_^2^ + 2*F*_c_^2^)/3
1577 reflections	(Δ/σ)_max_ < 0.001
106 parameters	Δρ_max_ = 0.31 e Å^−^^3^
0 restraints	Δρ_min_ = −0.39 e Å^−^^3^

### Special details

**Table:**

Geometry. All e.s.d.'s (except the e.s.d. in the dihedral angle between two l.s. planes) are estimated using the full covariance matrix. The cell e.s.d.'s are taken into account individually in the estimation of e.s.d.'s in distances, angles and torsion angles; correlations between e.s.d.'s in cell parameters are only used when they are defined by crystal symmetry. An approximate (isotropic) treatment of cell e.s.d.'s is used for estimating e.s.d.'s involving l.s. planes.
Refinement. Refinement of *F*^2^ against ALL reflections. The weighted *R*-factor *wR* and goodness of fit *S* are based on *F*^2^, conventional *R*-factors *R* are based on *F*, with *F* set to zero for negative *F*^2^. The threshold expression of *F*^2^ > σ(*F*^2^) is used only for calculating *R*-factors(gt) *etc*. and is not relevant to the choice of reflections for refinement. *R*-factors based on *F*^2^ are statistically about twice as large as those based on *F*, and *R*- factors based on ALL data will be even larger.

### Fractional atomic coordinates and isotropic or equivalent isotropic displacement parameters (Å^2^)

**Table:**

	*x*	*y*	*z*	*U*_iso_*/*U*_eq_	
Pd1	0.0000	0.0000	0.0000	0.01570 (9)	
Cl1	−0.30064 (12)	−0.26378 (11)	0.02745 (2)	0.02254 (15)	
N1	−0.1173 (4)	0.2075 (4)	0.05318 (8)	0.0188 (5)	
H1A	−0.162 (5)	0.332 (5)	0.0398 (9)	0.019 (7)*	
H1B	−0.282 (6)	0.151 (5)	0.0615 (10)	0.029 (8)*	
O1	0.6985 (4)	0.2939 (4)	0.21008 (7)	0.0347 (5)	
C1	0.0967 (5)	0.2336 (4)	0.09336 (9)	0.0191 (5)	
C2	0.2570 (5)	0.4248 (4)	0.09767 (9)	0.0202 (6)	
H2	0.2259	0.5402	0.0744	0.024*	
C3	0.4636 (5)	0.4498 (4)	0.13583 (9)	0.0231 (6)	
H3	0.5742	0.5818	0.1387	0.028*	
C4	0.5079 (5)	0.2813 (5)	0.16982 (9)	0.0251 (6)	
C5	0.3517 (6)	0.0864 (5)	0.16452 (10)	0.0290 (6)	
H5	0.3861	−0.0310	0.1872	0.035*	
C6	0.1466 (6)	0.0617 (4)	0.12644 (10)	0.0256 (6)	
H6	0.0401	−0.0722	0.1229	0.031*	
C7	0.8612 (6)	0.4926 (6)	0.21595 (11)	0.0395 (7)	
H7A	0.9714	0.5130	0.1879	0.059*	
H7B	0.9903	0.4817	0.2454	0.059*	
H7C	0.7340	0.6199	0.2186	0.059*	

### Atomic displacement parameters (Å^2^)

**Table:**

	*U*^11^	*U*^22^	*U*^33^	*U*^12^	*U*^13^	*U*^23^
Pd1	0.01623 (13)	0.01298 (15)	0.01751 (14)	−0.00122 (12)	−0.00092 (9)	−0.00012 (13)
Cl1	0.0220 (3)	0.0164 (3)	0.0292 (3)	−0.0035 (3)	0.0023 (2)	0.0032 (3)
N1	0.0196 (11)	0.0148 (12)	0.0217 (12)	0.0004 (10)	−0.0008 (9)	0.0007 (10)
O1	0.0334 (10)	0.0448 (14)	0.0237 (10)	0.0059 (10)	−0.0100 (8)	−0.0007 (10)
C1	0.0191 (12)	0.0210 (14)	0.0173 (12)	0.0030 (11)	0.0029 (10)	−0.0047 (12)
C2	0.0245 (13)	0.0187 (14)	0.0177 (13)	0.0003 (11)	0.0040 (10)	0.0011 (11)
C3	0.0221 (12)	0.0239 (16)	0.0230 (14)	−0.0030 (11)	0.0009 (10)	−0.0037 (12)
C4	0.0222 (13)	0.0359 (18)	0.0171 (13)	0.0067 (13)	0.0000 (10)	−0.0053 (13)
C5	0.0380 (16)	0.0260 (15)	0.0226 (15)	0.0066 (13)	−0.0005 (12)	0.0049 (13)
C6	0.0329 (14)	0.0191 (15)	0.0245 (14)	0.0004 (12)	0.0013 (11)	−0.0004 (12)
C7	0.0320 (15)	0.055 (2)	0.0303 (15)	−0.0001 (17)	−0.0054 (12)	−0.0113 (17)

### Geometric parameters (Å, °)

**Table:**

Pd1—N1	2.042 (2)	C2—C3	1.387 (3)
Pd1—N1^i^	2.042 (2)	C2—H2	0.9500
Pd1—Cl1	2.3010 (6)	C3—C4	1.387 (4)
Pd1—Cl1^i^	2.3010 (6)	C3—H3	0.9500
N1—C1	1.449 (3)	C4—C5	1.386 (4)
N1—H1A	0.85 (3)	C5—C6	1.380 (4)
N1—H1B	0.90 (3)	C5—H5	0.9500
O1—C4	1.376 (3)	C6—H6	0.9500
O1—C7	1.422 (4)	C7—H7A	0.9800
C1—C2	1.376 (4)	C7—H7B	0.9800
C1—C6	1.387 (4)	C7—H7C	0.9800
			
N1—Pd1—N1^i^	180.00 (8)	C2—C3—C4	119.7 (2)
N1—Pd1—Cl1	88.23 (7)	C2—C3—H3	120.2
N1^i^—Pd1—Cl1	91.77 (7)	C4—C3—H3	120.2
N1—Pd1—Cl1^i^	91.77 (7)	O1—C4—C5	116.1 (3)
N1^i^—Pd1—Cl1^i^	88.23 (7)	O1—C4—C3	124.1 (3)
Cl1—Pd1—Cl1^i^	180.0	C5—C4—C3	119.7 (2)
C1—N1—Pd1	113.89 (15)	C6—C5—C4	120.4 (3)
C1—N1—H1A	111.6 (18)	C6—C5—H5	119.8
Pd1—N1—H1A	107.2 (17)	C4—C5—H5	119.8
C1—N1—H1B	114.1 (17)	C5—C6—C1	119.6 (3)
Pd1—N1—H1B	104.4 (18)	C5—C6—H6	120.2
H1A—N1—H1B	105 (2)	C1—C6—H6	120.2
C4—O1—C7	116.8 (2)	O1—C7—H7A	109.5
C2—C1—C6	120.1 (2)	O1—C7—H7B	109.5
C2—C1—N1	120.1 (2)	H7A—C7—H7B	109.5
C6—C1—N1	119.7 (2)	O1—C7—H7C	109.5
C1—C2—C3	120.3 (2)	H7A—C7—H7C	109.5
C1—C2—H2	119.8	H7B—C7—H7C	109.5
C3—C2—H2	119.8		
			
Cl1—Pd1—N1—C1	105.56 (18)	C7—O1—C4—C3	−1.1 (4)
Cl1^i^—Pd1—N1—C1	−74.44 (18)	C2—C3—C4—O1	−177.0 (2)
Pd1—N1—C1—C2	103.7 (2)	C2—C3—C4—C5	2.0 (4)
Pd1—N1—C1—C6	−73.9 (2)	O1—C4—C5—C6	177.2 (2)
C6—C1—C2—C3	−1.9 (4)	C3—C4—C5—C6	−1.9 (4)
N1—C1—C2—C3	−179.5 (2)	C4—C5—C6—C1	−0.1 (4)
C1—C2—C3—C4	−0.2 (4)	C2—C1—C6—C5	2.0 (4)
C7—O1—C4—C5	179.8 (2)	N1—C1—C6—C5	179.6 (2)

Symmetry codes: (i) −*x*, −*y*, −*z*.

### Hydrogen-bond geometry (Å, °)

**Table:**

*D*—H···*A*	*D*—H	H···*A*	*D*···*A*	*D*—H···*A*
N1—H1A···Cl1^ii^	0.85 (3)	2.53 (3)	3.353 (2)	162 (2)

Symmetry codes: (ii) *x*, *y*+1, *z*.

## References

[bb1] Bon, V., Dudko, A., Orysyk, S. & Pekhnyo, V. (2009). *Acta Cryst.* E**65**, m396. 10.1107/S1600536809008472 PMC2968883 21582343

[bb2] Bruker (2005). *APEX2*, *SAINT* and *SADABS* Bruker AXS Inc., Madison, Wisconsin, USA.

[bb3] Casas, J. S., Castellano, E. E., Ellena, J., García-Tasende, M. S., Pérez-Parallé, M. L., Sánchez, A., Sánchez-González, A. & Touceda, A. (2008). *J. Inorg. Biochem.* **102**, 33–45. 10.1016/j.jinorgbio.2007.06.032 17689616

[bb4] Ojwach, S. O., Westman, G. & Darkwa, J. (2007). *Polyhedron*, **26**, 5544–5552.

[bb5] Pan, Y.-L., Zhao, F. & Yang, S. (2006). *Acta Cryst.* E**62**, m239–m240.

[bb6] Sheldrick, G. M. (2008). *Acta Cryst.* A**64**, 112–122. 10.1107/S0108767307043930 18156677

[bb7] Westrip, S. P. (2009). *publCIF.* In preparation.

